# Supplementary material for: Cost effectiveness and return on investment analysis for surgical care in a conflict-affected region of Sudan
Source: PLOS Glob Public Health. 2024 Nov 4;4(11):e0003712. doi: 10.1371/journal.pgph.0003712 (PMC11534226; doi:10.1371/journal.pgph.0003712)
Supplement: S1 Appendix — Table A. Disability Weight Assignments. Table B. Probability of Successful Treatment Assignments. Table C. Risk of Death Assignments. Table D. Cost-Effectiveness Ratios Across Surgical Specialties. Table E. One Way Deterministic Sensitivity Analysis for Cost-Effectiveness Ratios for Surgical Care at Mother of Mercy Hospital. Table F. One Way Deterministic Sensitivity Analysis for Societal Economic Impact of Surgical Care at Mother of Mercy Hospital. Table G. Two Way Deterministic Sensitivity Analysis for Cost-Effectiveness of Surgical Care at Mother of Mercy Hospital. Table H. Two Way Deterministic Sensitivity Analysis for Societal Economic Impact of Surgical Care at Mother of Mercy Hospital. Table I. References for Appendices Tables. Table J. Author Reflexivity Statement. (DOCX) [file pgph.0003712.s002.docx]

| **Table A: Disability Weight Assignments** | | |
| --- | --- | --- |
| **Diagnosis** | **DW** | **Reference** |
| Cataract | 0.184 | GBD 2019, severe vision impairment due to cataract |
| Abscess | 0.006 | GBD 2019, abscess and other bacterial skin diseases |
| Dental | 0.006 | GBD 2019, mild other oral disorders |
| Hernia | 0.114; 0.324 | GBD 2019, moderate symptomatic inguinal, femoral and abdominal hernia; severe symptomatic inguinal, femoral, and abdominal hernia |
| Cesarean section | 0.324 | GBD 2019, obstructed labor, acute event |
| Miscarriage / Blighted Ovum | 0.114 | GBD 2019, maternal abortive outcome |
| Necrotic Wound / Gangrene | 0.011 | GBD 2019, symptomatic other skin and soft tissue diseases |
| Foreign body | 0.011 | GBD 2019, symptomatic other skin and soft tissue diseases |
| Laceration | 0.011 | GBD 2019, symptomatic other skin and soft tissue diseases |
| Foreign body, eye | 0.031 | GBD 2019, GBD 2019, distance vision moderate impairment |
| Hydrocele | 0.011 | GBD 2019, abdominopelvic problem, mild |
| Lipoma | 0.011 | GBD 2019, symptomatic other skin and soft tissue diseases |
| Fracture / Non-Union | 0.228 | Femur fracture, Haagsma et al. |
| Burn | 0.011; 0.264 | GBD 2019, symptomatic other skin and soft tissue diseases; 5-20% TBSA Burn, Spronk et al. |
| Benign prostatic hypertrophy | 0.114 | GBD 2019, abdominopelvic problem, moderate |
| Urologic stricture | 0.114 | GBD 2019, abdominopelvic problem, moderate |
| Chronic osteomyelitis | 0.053 | Haagsma et al. |
| Ear Pain/Wax/Pus | 0.013 | GBD 2019, ear pain |
| Anal fissure | 0.082 | Haagsma et al. |
| Urologic stone | 0.114 | GBD 2019, moderate urolithiasis episodes |
| Implant | 0.011 | GBD 2019, symptomatic other skin and soft tissue diseases |
| Ectopic pregnancy | 0.114 | GBD 2019, ectopic pregnancy |
| Subcutaneous mass | 0.011 | GBD 2019, symptomatic other skin and soft tissue diseases |
| Vesicovaginal fistula | 0.342 | GBD 2019, vesicovaginal fistula |
| Goiter | 0.067 | GBD 2019, disfigurement level 2 |
| Hydrocephalus | 0.402 | GBD 2019, severe motor impairment due to neonatal encephalopathy due to birth asphyxia and trauma |
| Breast Cancer/Fibroadenoma | 0.288; 0.011 | GBD 2019, diagnosis and primary therapy phase of breast cancer; symptomatic other skin and soft tissue diseases |
| Unknown Mass/Lymphadenopathy | 0.011 | GBD 2019, symptomatic other skin and soft tissue diseases |
| Appendicitis | 0.324 | GBD 2019, appendicitis |
| Perianal Fistula | 0.082 | Haagsma et al. |
| Circumcision | 0.028 | Saxton et al. |
| Prostate cancer | 0.288 | GBD 2019, diagnosis and primary therapy phase of prostate cancer |
| Colorectal/Anorectal cancer | 0.288 | GBD 2019, diagnosis and primary therapy phase of colon and rectal cancers |
| Fibroid uterus | 0.062 | GBD 2019, mild abdominal pain due to uterine fibroids withj moderate anemia |
| Extremity condition requiring amputation | 0.282 | GBD 2019, diabetic neuropathy with untreated amputation |
| Small bowel obstruction | 0.324 | GBD 2019, severe abdominopelvic problem |
| Urinary retention | 0.114 | GBD 2019, abdominopelvic problem, moderate |

| **Table B: Probability of Successful Treatment Assignments** | | |
| --- | --- | --- |
| **Diagnosis** | **PST** | **Reference** |
| Cataract | 0.875 | Ruit et al. and Liu et al. |
| Abscess | 0.92; 0.96 | Talan et al. |
| Dental | 1 | Assumption |
| Hernia | 0.98; 0.96 | 1.9% recurrence for open non-mesh elective hernia repairs, HerniaSurge Group; 4% recurrence for open non-mesh emergent repair, Nielson et al. |
| Cesarean section | 1 | Assumption |
| Miscarriage / Blighted Ovum | 1 | Assumption |
| Necrotic Wound / Gangrene | 0.708 | Wilcox et al. |
| Foreign body | 1 | Assumption |
| Laceration | 1 | Assumption |
| Foreign body, eye | 0.98 | Assumption |
| Hydrocele | 0.96 | Mante et al. |
| Lipoma | 0.99 | Weiss et al. |
| Fracture / Non-Union | 0.977 | Per MMH re-operation rate in 2022 |
| Burn | 0.9 | Herndon et al. |
| Benign prostatic hypertrophy | 0.8 | Kim et al. |
| Urologic stricture | 0.65 | Steenkamp et al. |
| Chronic osteomyelitis | 0.8 | Geurts et al. |
| Ear Pain/Wax/Pus | 1 | Assumption |
| Anal fissure | 0.92 | Davies et al. |
| Urologic stone | 0.95 | Assumption |
| Implant | 1 | Assumption |
| Ectopic pregnancy | 1 | Assumption |
| Subcutaneous mass | 0.99 | Weiss et al. |
| Vesicovaginal fistula | 0.95 | Maroyi et al. |
| Goiter | 0.97 | Attaallah et al. |
| Hydrocephalus | 0.268 | Reddy et al. |
| Breast Cancer/Fibroadenoma | 0.7; 1 | Assumption |
| Unknown Mass/Lymphadenopathy | 0.99 | Weiss et al. |
| Appendicitis | 0.98 | Podda et al. |
| Perianal Fistula | 0.899 | Abbas et al. |
| Circumcision | 1 | Assumption |
| Prostate cancer | 0.7 | Assumption |
| Colorectal/Anorectal cancer | 0.8 | Assumption |
| Fibroid uterus | 0.98 | Assumption |
| Extremity condition requiring amputation | 1 | Assumption |
| Small bowel obstruction | 0.7 | Assumption |
| Urinary retention | 0.9 | Assumption |

| **Table C: Risk of Death Assignments** | | | | |
| --- | --- | --- | --- | --- |
| **Diagnosis** | **RD** | **Remaining lifespan lost to death without treatment** | **Remaining lifespan disabled without treatment** | **Reference** |
| Cataract | 0 | 0 | 1 | Assumption, with reference to Gosselin et al. and Chatterjee et al. |
| Abscess | 0 | 0 | 0.05 | Assumption, with reference to Gosselin et al. and Chatterjee et al. |
| Dental | 0 | 0 | 1 | Assumption, with reference to Gosselin et al. and Chatterjee et al. |
| Hernia | 0.07 - 1 | 0.10-0.99 | 0.01-0.90 | Elective: Risk of incarceration per Gallegos et al.; Incarcerated or Strangulated: Assumption, per Gosselin et al. and Chatterjee et al. |
| Cesarean section | 1 | 0.99 | 0.01 | Assumption, with reference to Gosselin et al. and Chatterjee et al. |
| Miscarriage / Blighted Ovum | 0 | 0 | 1 | Assumption, with reference to Gosselin et al. and Chatterjee et al. |
| Necrotic Wound / Gangrene | 0.3 | 0.99 | 0.01 | Assumption, with reference to Gosselin et al. and Chatterjee et al. |
| Foreign body | 0 | 0 | 1 | Assumption, with reference to Gosselin et al. and Chatterjee et al. |
| Laceration | 0 | 0 | 0.1 | Assumption, with reference to Gosselin et al. and Chatterjee et al. |
| Foreign body, eye | 0 | 0 | 1 | Assumption, with reference to Gosselin et al. and Chatterjee et al. |
| Hydrocele | 0 | 0 | 0.2 | Assumption, with reference to Gosselin et al. and Chatterjee et al. |
| Lipoma | 0 | 0 | 1 | Assumption, with reference to Gosselin et al. and Chatterjee et al. |
| Fracture / Non-Union | 0.7 | 0.5 | 0.5 | Assumption, with reference to Gosselin et al. and Chatterjee et al. |
| Burn | 0.7 | 0.99 | 0.01 | Assumption, with reference to Gosselin et al. and Chatterjee et al. |
| Benign prostatic hypertrophy | 0 | 0 | 1 | Assumption, with reference to Gosselin et al. and Chatterjee et al. |
| Urologic stricture | 0.3 | 0.5 | 0.5 | Assumption, with reference to Gosselin et al. and Chatterjee et al. |
| Chronic osteomyelitis | 0.3 | 0.9 | 0.1 | Assumption, with reference to Gosselin et al. and Chatterjee et al. |
| Ear Pain/Wax/Pus | 0 | 0 | 0.05 | Assumption, with reference to Gosselin et al. and Chatterjee et al. |
| Anal fissure | 0 | 0 | 1 | Assumption, with reference to Gosselin et al. and Chatterjee et al. |
| Urologic stone | 0.7 | 0.9 | 0.1 | Assumption, with reference to Gosselin et al. and Chatterjee et al. |
| Implant | 0 | 0 | 1 | Assumption, with reference to Gosselin et al. and Chatterjee et al. |
| Ectopic pregnancy | 0.75 | 0.99 | 0.01 | Shrime et al. |
| Subcutaneous mass | 0 | 0 | 1 | Assumption, with reference to Gosselin et al. and Chatterjee et al. |
| Vesicovaginal fistula | 0.7 | 0.9 | 0.1 | Assumption, with reference to Gosselin et al. and Chatterjee et al. |
| Goiter | 0.1 | 0.2 | 0.8 | Assumption, with reference to Gosselin et al. and Chatterjee et al. |
| Hydrocephalus | 0.7 | 0.9 | 0.1 | Assumption, with reference to Gosselin et al. and Chatterjee et al. |
| Breast Cancer/Fibroadenoma | 0 - 0.7 | 0-0.80 | 0.2-1 | Assumption, with reference to Gosselin et al. and Chatterjee et al. |
| Unknown Mass/Lymphadenopathy | 0 | 0 | 1 | Assumption, with reference to Gosselin et al. and Chatterjee et al. |
| Appendicitis | 1 | 0.99 | 0.01 | Assumption, with reference to Gosselin et al. and Chatterjee et al. |
| Perianal Fistula | 0 | 0 | 1 | Assumption, with reference to Gosselin et al. and Chatterjee et al. |
| Circumcision | 0 | 0 | 1 | Assumption, with reference to Gosselin et al. and Chatterjee et al. |
| Prostate cancer | 0.7 | 0.1 | 0.9 | Assumption, with reference to Gosselin et al. and Chatterjee et al. |
| Colorectal/Anorectal cancer | 0.7 | 0.9 | 0.1 | Assumption, with reference to Gosselin et al. and Chatterjee et al. |
| Fibroid uterus | 0.3 | 0.2 | 0.8 | Assumption, with reference to Gosselin et al. and Chatterjee et al. |
| Extremity condition requiring amputation | 0.75 | 0.99 | 0.01 | Abbas and Archibald |
| Small bowel obstruction | 0.3 | 0.99 | 0.01 | Assumption, with reference to Gosselin et al. and Chatterjee et al. |
| Urinary retention | 0.7 | 0 | 1 | Assumption, with reference to Gosselin et al. and Chatterjee et al. |

| **Table D: Cost-Effectiveness Ratios Across Surgical Specialties** | | |
| --- | --- | --- |
| **Specialty** | **Number of procedures** | **Average CER ($/DALY Averted)** |
| General surgery | 994 | 174.14 |
| Neurosurgery | 14 | 40.58 |
| Obstetrics and Gynecology | 292 | 15.70 |
| Ophthalmology | 679 | 674.16 |
| Oral and maxillofacial surgery | 229 | 5,495.67 |
| Orthopedic surgery | 79 | 21.41 |
| Plastic surgery | 55 | 19.77 |
| Urology | 93 | 66.81 |
| **Total** | **2435** | **72.54** |
| Average cost-effectiveness ratios for surgical care at Mother of Mercy Hospital across different surgical specialties. CER-Cost-effectiveness ratio; DALY-Disability adjusted life year. | | |

| **Table E: One Way Deterministic Sensitivity Analysis for Cost-Effectiveness Ratios for Surgical Care at Mother of Mercy Hospital** | | | |
| --- | --- | --- | --- |
|  | **Average Cost-Effectiveness Ratio** | | |
| **Variable** | **Baseline** | **Lower Bound** | **Upper Bound** |
| No 3% discounting | $72.54 | $39.66 | $72.54 |
| Extrapolation | $72.54 | $61.66 | $83.43 |
| Proportion of beds attributed to surgery | $72.54 | $62.49 | $82.60 |
| Risk of death | $72.54 | $68.43 | $84.54 |
| Probability of successful treatment | $72.54 | $69.62 | $85.35 |
| Age-weighting | $72.54 | $64.39 | $72.54 |
| Predicted age of death without treatment | $72.54 | $71.44 | $78.40 |
| Disability weight | $72.54 | $71.94 | $73.16 |
| Equipment value | $72.54 | $72.27 | $72.82 |
| Duration of disability | $72.54 | $72.54 | $72.95 |
| Property value | $72.54 | $72.47 | $72.62 |
| Probability of complications | $72.54 | $72.53 | $72.56 |
| Complication disability weight | $72.54 | $72.53 | $72.56 |
| Baseline costs and disability-adjusted life years (DALY) averted were calculated using 3% discounting and no age-weighting (3,0). Individual variables were tested for impact on the average cost-effectiveness ratio. | | | |

| **Table F: One Way Deterministic Sensitivity Analysis for Societal Economic Impact of Surgical Care at Mother of Mercy Hospital** | | | |
| --- | --- | --- | --- |
|  | **Total Societal Economic Benefit** | | |
| **Variable** | **Baseline** | **Lower Bound** | **Upper Bound** |
| No 3% discounting | $9,124,686 | $9,124,686 | $16,691,318 |
| Extrapolation | $9,124,686 | $7,934,509 | $10,734,925 |
| Risk of death | $9,124,686 | $7,830,321 | $9,673,546 |
| Probability of successful treatment | $9,124,686 | $7,755,983 | $9,508,012 |
| Age weighting | $9,124,686 | $9,124,686 | $10,279,725 |
| Predicted age of death without treatment | $9,124,686 | $8,442,831 | $9,265,229 |
| Disability weight | $9,124,686 | $9,048,286 | $9,201,086 |
| Duration of disability | $9,124,686 | $9,073,906 | $9,125,025 |
| Probability of complications | $9,124,686 | $9,122,624 | $9,126,748 |
| Complication disability weight | $9,124,686 | $9,122,624 | $9,126,748 |
| Baseline societal economic benefit calculated by multiplying gross national income per capita in South Sudan ($1,040) by total disability-adjusted life years (DALY) averted calculated using 3% discounting and no age-weighting (3,0). Individual variables were tested for impact on the overall societal economic benefit through changes in DALY averted. | | | |

| **Table G: Two Way Deterministic Sensitivity Analysis for Cost-Effectiveness of Surgical Care at Mother of Mercy Hospital** | | | | |
| --- | --- | --- | --- | --- |
|  |  | **Average Cost-Effectiveness Ratio** | | |
| **Surgical specialty** | **Number of procedures** | **Baseline** | **Minimum** | **Maximum** |
| General surgery | 994 | 174.14 | 70.7 | 301.91 |
| Neurosurgery | 14 | 40.58 | 11.35 | 68.78 |
| Obstetrics and Gynecology | 292 | 15.7 | 6.77 | 26.85 |
| Ophthalmology | 679 | 674.16 | 309.29 | 1,200.27 |
| Oral and maxillofacial surgery | 229 | 5,495.67 | 2,219.44 | 9,452.64 |
| Orthopedic surgery | 79 | 21.41 | 8.95 | 37.61 |
| Plastic surgery | 55 | 19.77 | 6.45 | 33.62 |
| Urology | 93 | 66.81 | 26.41 | 117.08 |
| **Total from sampled cohort** | **2,435** | **72.54** | **29.83** | **124.93** |
| **Total after extrapolation** | **3,016** | **72.54** | **25.36** | **143.67** |

| **Table H: Two Way Deterministic Sensitivity Analysis for Societal Economic Impact of Surgical Care at Mother of Mercy Hospital** | | | | |
| --- | --- | --- | --- | --- |
|  |  | **Total Economic Impact** | | |
| **Surgical specialty** | **Number of surgeries** | **Baseline** | **Minimum** | **Maximum** |
| General surgery | 994 | $1,252,787 | $826,725 | $2,645,519 |
| Neurosurgery | 14 | $75,721 | $51,114 | $232,180 |
| Obstetrics and Gynecology | 292 | $4,082,313 | $2,730,545 | $8,118,878 |
| Ophthalmology | 679 | $221,055 | $142,050 | 413,094.00 |
| Oral and maxillofacial surgery | 229 | 9,145.00 | 6,083.00 | 19,415.00 |
| Orthopedic surgery | 79 | $809,756 | $527,426 | $1,661,477 |
| Plastic surgery | 55 | $610,633 | $410,767 | $1,604,210 |
| Urology | 93 | $305,503 | $199,457 | $662,612 |
| Total from sampled cohort | 2,435 | $7,366,913 | $4,894,166 | $15,357,386 |
| Total after extrapolation | 3,016 | $9,124,686 | $5,271,246 | $22,378,488 |

| **Table I: References for Appendices Tables** |
| --- |
| Abbas MA, Jackson CH, Haigh PI. Predictors of outcome for anal fistula surgery. Arch Surg. 2011;146(9):1011-1016. |
|  |
| Alemu A, Yadeta E, Deressa A, et al. Survival status and predictors of mortality among women with uterine rupture at public hospitals of Eastern Ethiopia. Semi-parametric survival analysis. Int J Womens Health. 2023;15:443-453. |
|  |
| Ali Y. Analysis of Cesarean delivery in Jimma Hospital, south-western Ethiopia. East Afr Med J. 1995;72(1):60-63. |
|  |
| Attaallah W, Erel S, Canturk NZ, et al. Is hemithyroidectomy a rational management for benign nodular goitre? A multicentre retrospective single group study. Neth J Med. 2015;73(1):17-22. |
|  |
| Bay-Nielsen M, Kehlet H, Strand L, et al. Quality assessment of 26 304 herniorrhaphies in Denmark: A prospective nationwide study. Lancet. 2001;358(9288):1124-1128. |
|  |
| Chatterjee S, Laxminarayan R, Gosselin RA. Cost Per DALY Averted in a Surgical Unit of a Private Hospital in India. World J Surg. 2016;40(5):1034-1040. |
|  |
| Cordeiro E, Jackson TD, Elnahas A, Cil T. Higher rate of breast surgery complications in patients with metastatic breast cancer: An analysis of the NSQIP database. Ann Surg Oncol. 2014;21(10):3167-3172. |
|  |
| Davies I, Dafydd L, Davies L, Beynon J. Long term outcomes after lateral anal sphincterotomy for anal fissure: a retrospective cohort study. Surg Today. 2014;44(6):1032-1039. |
|  |
| Eryilmaz R, Sahin M, Hakan Tekelioglu M, Daldal E. Management of lactational breast abscesses. The Breast. 2005;14(5):375-379. |
|  |
| Fishel Bartal M, Sibai BM. Eclampsia in the 21st century. Am J Obstet Gynecol. 2022;226(2S):S1237-S1253. |
|  |
| Gallegos NC, Dawson J, Jarvis M, Hobsley M. Risk of strangulation in groin hernias. Br J Surg. 1991;78(10):1171-1173. |
|  |
| Geurts J, Hohnen A, Vranken T, Moh P. Treatment strategies for chronic osteomyelitis in low- and middle-income countries: systematic review. Tropical Medicine & International Health. 2017;22(9):1054-1062. |
|  |
| Global Burden of Disease Collaborative Network. Global Burden of Disease Study 2019 (GBD 2019) Disability Weights. Institute for Health Metrics and Evaluation (IHME), 2020.<https://doi.org/10.6069/1W19-VX76>. |
|  |
| Gosselin RA, Maldonado A, Elder G. Comparative cost-effectiveness analysis of two MSF surgical trauma centers. World J Surg. 2010;34(3):415-419. |
|  |
| Gupta NP, Kochar GS, Wadhwa SN, Singh SM. Management of patients with renal and ureteric calculi presenting with chronic renal insufficiency. Br J Urol. 1985;57(2):130-132. |
|  |
| Gupta PK, Smith RB, Gupta H, Forse RA, Fang X, Lydiatt WM. Outcomes after thyroidectomy and parathyroidectomy. Head Neck. 2012;34(4):477-484. doi:10.1002/hed.21757 |
|  |
| Hanak BW, Bonow RH, Harris CA, Browd SR. Cerebrospinal fluid shunting complications in children. Pediatr Neurosurg. 2017;52(6):381-400. |
|  |
| Harris AM, Althausen PL, Kellam J, Bosse MJ, Castillo R. Lower Extremity Assessment Project (LEAP) Study Group. Complications following limb-threatening lower extremity trauma. J Orthop Trauma. 2009;23(1):1-6. doi:10.1097/BOT.0b013e31818e43dd |
|  |
| Herndon DN, Barrow RE, Rutan RL, Rutan TC, Desai MH, Abston S. A comparison of conservative versus early excision. Therapies in severely burned patients. Ann Surg. 1989;209(5):547-553. |
|  |
| HerniaSurge Group. International guidelines for groin hernia management. Hernia. 2018;22(1):1-165. |
|  |
| Ingraham AM, Cohen ME, Bilimoria KY, Pritts TA, Ko CY, Esposito TJ. Comparison of outcomes after laparoscopic versus open appendectomy for acute appendicitis at 222 ACS NSQIP hospitals. Surgery. 2010;148(4):625-635; discussion 635-637. |
|  |
| Kim EH, Larson JA, Andriole GL. Management of benign prostatic hyperplasia. Annu Rev Med. 2016;67:137-51. |
|  |
| Kumbargere Nagraj S, Prashanti E, Aggarwal H, et al. Interventions for treating post-extraction bleeding. Cochrane Database Syst Rev. 2018;3(3):CD011930. |
|  |
| Kwaan MR, Al-Refaie WB, Parsons HM, Chow CJ, Rothenberger DA, Habermann EB. Are right-sided colectomy outcomes different from left-sided colectomy outcomes?: study of patients with colon cancer in the ACS NSQIP database. JAMA Surg. 2013;148(6):504-510. |
|  |
| Lombardo S, Baum K, Filho JD, Nirula R. Should adhesive small bowel obstruction be managed laparoscopically? A National Surgical Quality Improvement Program propensity score analysis. J Trauma Acute Care Surg. 2014 Mar;76(3):696-703. |
|  |
| Mante SD, Gueye SM. Capacity building for the modified filarial hydrocelectomy technique in West Africa. Acta Trop. 2011;120 Suppl 1:S76-80. |
|  |
| Maroyi R, Shahid U, Vangaveti V, Rane A, Mukwege D. Obstetric vesico-vaginal fistulas: Midvaginal and juxtacervical fistula repair outcomes in the Democratic Republic of Congo. Int J Gynaecol Obstet. 2021;153(2):294-299. |
|  |
| Nanda K, Lopez LM, Grimes DA, Peloggia A, Nanda G. Expectant care versus surgical treatment for miscarriage. Cochrane Database Syst Rev. 2012;2012(3):CD003518. doi:10.1002/14651858.CD003518.pub3 |
|  |
| Podda M, Cillara N, Di Saverio S, et al. Antibiotics-first strategy for uncomplicated acute appendicitis in adults is associated with increased rates of peritonitis at surgery. A systematic review with meta-analysis of randomized controlled trials comparing appendectomy and non-operative management with antibiotics. Surgeon. 2017;15(5):303-314. |
|  |
| Rami Reddy SR, Cappell MS. A systematic review of the clinical presentation, diagnosis, and treatment of small bowel obstruction. Curr Gastroenterol Rep. 2017;19(6):28. |
|  |
| Reddy GK, Bollam P, Caldito G. Long-term outcomes of ventriculoperitoneal shunt surgery in patients with hydrocephalus. World Neurosurg. 2014;81(2):404-410. |
|  |
| Sahnan K, Adegbola SO, Tozer PJ, Watfah J, Phillips RK. Perianal abscess. BMJ. 2017;356:j475. |
|  |
| Shrime MG, Verguet S, Johansson KA, Desalegn D, Jamison DT, Kruk ME. Task-sharing or public finance for the expansion of surgical access in rural Ethiopia: an extended cost-effectiveness analysis. Health Policy and Planning. 2016;31(6):706-716. |
|  |
| Steenkamp JW, Heyns CF, de Kock ML. Internal urethrotomy versus dilation as treatment for male urethral strictures: A prospective, randomized comparison. J Urol. 1997 Jan;157(1):98-101. |
|  |
| Stewart KA, Tessier KM, Lebovic DI. Comparing characteristics of and postoperative morbidity after hysterectomy for endometriosis versus other benign indications: A NSQIP study. J Minim Invasive Gynecol. 2022 Jul;29(7):884-890.e2. |
|  |
| Talan DA, Mower WR, Krishnadasan A, et al. Trimethoprim-sulfamethoxazole versus placebo for uncomplicated skin abscess. N Engl J Med. 2016;374(9):823-832. |
|  |
| Thonneau P, Hijazi Y, Goyaux N, Calvez T, Keita N. Ectopic pregnancy in Conakry, Guinea. Bull World Health Organ. 2002;80(5):365-370. |
|  |
| Weiss HA, Larke N, Halperin D, Schenker I. Complications of circumcision in male neonates, infants and children: a systematic review. BMC Urol. 2010;10:2. |
|  |
| Weiss LM. Soft Tissues. In: Weidner N, Cote RJ, Suster S, Weiss LM, eds. Modern Surgical Pathology (Second Edition). W.B. Saunders; 2009:1717-1783. |
|  |
| Wilcox JR, Carter MJ, Covington S. Frequency of debridements and time to heal: A retrospective cohort study of 312 744 wounds. JAMA Dermatol. 2013;149(9):1050-1058. |
|  |
| Xia L, Guzzo TJ, Mucksavage P, Lee DJ. Suprapubic versus urethral catheter for urinary drainage after robot-assisted radical prostatectomy. Curr Urol Rep. 2020;21(8):30. |

**Table J: Author Reflexivity Statement**

(Adapted from Morton, B., et al. Consensus statement on measures to promote equitable authorship in the publication of research from international partnerships. Anaesthesia, 2022;77(3):264-276.)

**Study conceptualisation**

**How does this study address local research and policy priorities?**

Healthcare in this conflict region of Sudan faces major challenges. This research addresses a critical research gap in the area, namely how can health care efficiently be delivered to the large at risk population in a cost-effective manner and one that can support local economies.

**How were local researchers involved in study design?**

The health leadership of the Mother of Mary Hospital were involved in all aspects of this research project, including initial study design, data collection, analysis, and writing. In discussions between researchers and hospital leadership, it was quickly recognized that the health system did not understand how efficiently their resources were being used for surgical care. All aspects of this project were designed to provide local health care leaders information to guide policy and resource allocation.

**Research management**

**How has funding been used to support the local research team(s)?**

No external funding was directly given to the local research team (nor any member of the research team). However, outside research experts donated their time and travel costs to oversee data collection and data analysis.

**Data acquisition and analysis**

**How are research staff who conducted data collection acknowledged?**

All members of the research staff who conducted data collection are included as co-authors in the manuscript.

**How have members of the research partnership been provided with access to study data?**

All members of the research partnership have access to all study data, analysis, and writing drafts. We used a secure password-protected Google Docs folder to allow data access to research partners throughout the research process.

**How were the data used to develop analytical skills within the partnership?**

As economic analyses were not common skills to many members of the research partnership, we used our Google Docs platform to share research methods, references, and other teaching tools such that all members of the research team learned these analytic skills.

**Data interpretation**

**How have research partners collaborated in interpreting study data?**

All research partners collaborated equally with data interpretation. We used our common Google Docs folder for development of all research results, tables, and figures. As summarized below, we used a common Google Docs manuscript to allow each research partner to offer their own input into data interpretation during drafting of the research manuscript.

**Drafting and revising for intellectual content**

**How were research partners supported to develop writing skills?**

As detailed above, all research partners were involved in drafting and editing of the research manuscript. The initial draft was written by trainees, and the senior author leads an annual workshop in scientific writing specifically for partners from LMICs, and many of the core principles in scientific writing (such as structure, style, and formatting) were used to guide all writing revisions.

**How will research products be shared to address local needs?**

The local hospital leadership as well as administrative leadership have already used these research findings to guide hospital strategic planning and discussions with local health ministry representatives. Administrators will continue to share these findings with new donors, who are key to sustainability of this health system.

**Authorship**

**How is the leadership, contribution and ownership of this work by LMIC researchers recognised within the authorship?**

Several of the leaders and co-authors are based in Sudan or Kenya, and their research involvement with this project has already advanced the research capacity at each of their home institutions.

**How have early career researchers across the partnership been included within the authorship team?**

Several members of the research partnership, including the lead author, are early stage researchers or trainees, including several co-authors who have not been involved in academic writing to date. All co-authors have been involved in the research process throughout the entire project, including project design, data acquisition, analysis, and manuscript preparation.

**How has gender balance been addressed within the authorship?**

All co-authors are members of the research partnership, with representation of both men and women in the partnership leading to gender balance in this project.

**Training**

**How has the project contributed to training of LMIC researchers?**

As described above, many members of the research team are trainees or early-stage researchers, including members both from LMICs as well as HIC. As such, the research capacity at all institutions has been advanced through this process.

**Infrastructure**

**How has the project contributed to improvements in local infrastructure?**

This project has identified the economic value of surgical care for the local hospital and health system. Although no local infrastructure expansion has been done directly to date as a result of this work, this project will be used to drive expansion of surgical services to donors and other funding agencies.

**Governance**

**What safeguarding procedures were used to protect local study participants and researchers?**

This work was reviewed by an IRB with all PHI removed from the records prior to data analysis. No PHI will be published. As this was an observational study involving economic analysis of deidentified data, there was no direct risk to patient or researchers.
