# Supplementary material for: Cost effectiveness and return on investment analysis for surgical care in a conflict-affected region of Sudan
Source: PLOS Glob Public Health. 2024 Nov 4;4(11):e0003712. doi: 10.1371/journal.pgph.0003712 (PMC11534226; doi:10.1371/journal.pgph.0003712)
Supplement: Attachment — Submitted filename: Resubmission.PLOS.GlobalHealth.Response to Reviewers.docx [file pgph.0003712.s003.docx]

Journal Requirements:

1. Please include a complete copy of PLOS’ questionnaire on inclusivity in global research in your revised manuscript. Our policy for research in this area aims to improve transparency in the reporting of research performed outside of researchers’ own country or community. The policy applies to researchers who have traveled to a different country to conduct research, research with Indigenous populations or their lands, and research on cultural artifacts. The questionnaire can also be requested at the journal’s discretion for any other submissions, even if these conditions are not met. Please find more information on the policy and a link to download a blank copy of the questionnaire here: https://journals.plos.org/plosone/s/best-practices-in-research-reporting. Please upload a completed version of your questionnaire as Supporting Information when you resubmit your manuscript.

**Please see the uploaded questionnaire as well as details of this information included within the Methods section of the text.**

Additional Editor Comments (if provided):

Needs major revision in view of strong criticism from one of the reviewers and other comments by 2nd reviewer

[Note: HTML markup is below. Please do not edit.]

Reviewers' comments:

Reviewer's Responses to Questions

Comments to the Author

1. Does this manuscript meet PLOS Global Public Health’s publication criteria? Is the manuscript technically sound, and do the data support the conclusions? The manuscript must describe methodologically and ethically rigorous research with conclusions that are appropriately drawn based on the data presented.

Reviewer #1: No

Reviewer #2: Yes

2. Has the statistical analysis been performed appropriately and rigorously?

Reviewer #1: Yes

Reviewer #2: Yes

3. Have the authors made all data underlying the findings in their manuscript fully available (please refer to the Data Availability Statement at the start of the manuscript PDF file)?

The PLOS Data policy requires authors to make all data underlying the findings described in their manuscript fully available without restriction, with rare exception. The data should be provided as part of the manuscript or its supporting information, or deposited to a public repository. For example, in addition to summary statistics, the data points behind means, medians and variance measures should be available. If there are restrictions on publicly sharing data—e.g. participant privacy or use of data from a third party—those must be specified.

Reviewer #1: Yes

Reviewer #2: Yes

4. Is the manuscript presented in an intelligible fashion and written in standard English?

PLOS Global Public Health does not copyedit accepted manuscripts, so the language in submitted articles must be clear, correct, and unambiguous. Any typographical or grammatical errors should be corrected at revision, so please note any specific errors here.

Reviewer #1: No

Reviewer #2: Yes

5. Review Comments to the Author

Please use the space provided to explain your answers to the questions above. You may also include additional comments for the author, including concerns about dual publication, research ethics, or publication ethics. (Please upload your review as an attachment if it exceeds 20,000 characters)

Reviewer #1: This paper assessed the socioeconomic cost-effectiveness of surgical care in the Nuba Mountains region of Sudan. The authors have performed a statistically rigorous analysis. However, I have several major concerns:

1. Sudan is presented as a “conflict setting” throughout the manuscript. However, this study uses data from 2022, under a ceasefire agreement. The authors do not mention the state the study takes place in, and mostly refer to the setting as “Sudan.” The data presented in this study is not from an active conflict setting. Conflict-affected may be a more appropriate terminology, although a clear description of what is meant would be required. Overall, a lack of clarity and even misleading description of the context is concerning.

**This reviewer brings up an important and relatively complicated point. We certainly did not mean to communicate that this data was collected during the most recent formally announced civil war, rather that the Nuba Mountains within the South Kordofan state of Sudan has been involved in chronic and cyclical conflict for decades. This region, which is overseen by a semi-autonomous, local rebel government, has essentially been excluded from the national government support, with associated long-term loss of public health infrastructure, high rates of internal displacement, and limited governance. The Uppsala Conflict Data Program has considered Sudan to be in a state of conflict since 1989 due to the chronic high numbers of inter-state, intrastate, non-state and one-sided deaths. Numerous sources have been cited in the paper have been used to support documentation of long-term impact of conflict both broadly across Sudan as well as within the relatively unique context of the Nuba Mountains.**

**We have substantially revised the text throughout to emphasize the long-term impacts of cyclical and chronic conflict on health care in the region. We appreciate the suggestion from this reviewer to use the term “conflict-affected,” and have also used the term “chronic conflict” throughout the text to emphasize these points. As well, we clarified the location of the study within the Nuba Mountains of the South Kordofan state of Sudan.**

2. Sudan was not in a civil war during 2022. Conflation with the current civil war throughout the paper is misleading.

**See above.**

3. The authors suggest local ethical oversight was not possible due to the ongoing conflict. While the data was generated during a ceasefire, data extraction took place during the current civil war (throughout 2023). However, it is not acceptable to have no local ethical oversight. Presumably, if local data extraction was possible, local ethical oversight would be as well. Research in active conflict settings has relied on community advisory boards. Other studies in Sudan have received ethical oversight from the General Directorate of Research of the Federal Ministry of Health.

**We appreciate the comment and are entirely supportive of the value of local oversight of ethical review for any research. However, there is simply no available national or regional IRB or community advisory boards in the Nuba Mountains region of Sudan, even prior to the most current formally announced national civil war. As summarized in the text and supported with citations, the Nuba Mountains region has been essentially excluded from any support or oversight from the central Directorate of Research or the Ministry of Health of the federal government for many years. In light of these limitations, we had both the hospital director as well as the Secretariat of Health of the local rebel government review this study prior to any data collection to ensure adherence to local ethical standards and ensure protection of patient confidentiality, and we added details on this local review within the text. We apologize for omitting this important information in the original version of our manuscript.**

4. Armed conflict “causes tremendous challenges to health systems” in countries of all economic classifications.

**We appreciate the comment and agree entirely. We certainly did not mean to communicate that conflict and its associated health impacts is limited to LMICs, and added clarification of the impact of conflict on all health systems and the need to understand economic impact in all wealth settings impacted by conflict.**

5. While the Discussion section mentions humanitarian reasons to provide surgical care, there is no mention of the right to health. It is unclear what international humanitarian law the authors reference (which is different that international human rights law).

**We appreciate this important comment on the value of human rights frameworks in support of the basic right to health for civilians in conflict settings as well as the distinctions between humanitarian law and human rights law. We agree entirely with these comments, and have added language emphasizing the basic rights to health as well as legal sources of these frameworks, such as codification within the Geneva Conventions.**

Reviewer #2: Little research has been conducted in conflict areas due to instability and logistical challenges conducting research in these settings. Research on these settings is limited, and there is an existing perception that surgery is too expensive in resource-limited settings. The authors should be applauded for their efforts and for shedding light on the cost-effectiveness of surgical services provision in resource-limited conflict areas.

The authors conducted a retrospective cohort study of 3,016 patients presenting over a 12 month period in a representative catchment hospital (Mother of Mercy-Gidel Hospital, a 425-bed hospital which is the only source of tertiary surgical care in the region). Staff is limited to a family physician trained in multiple surgical disciplines performing most of the surgical operations and two other physicians, mid-level ophthalmological provider, and mid-level dental provider, and three nurse anesthetists.

A single overall budget is used to track all hospital expenditures. This comprehensively assessed cost, including all direct medical costs for human resources/salaries, medications, disposables, and infrastructure as well as non-medical costs of patient and family meals. However, a micro-costing approach was not used, and for analysis of cost per case, they assumed the same costs for all procedures. Cost extrapolation was also based on commonly performed procedures (defined as >10 instances over the year), which was reasonable. Their sample of procedures each performed at least 10 times over the year represented 81% of all cases. Without clinical outcomes data, DALYs were modeled on predicted clinical outcomes.

The overall cost per surgical case was $211, average CER for all surgical care at MMH was $72.54/DALYa (less than Sudanese GDP/capita), determining CER is very cost-effective, further confirmed with appropriate sensitivity analyses. ROI was projected to be 14:1 for each dollar invested in surgical care in the study setting. Variability in CERs across surgical specialties was very interesting, with obstetrics and gynecology 367 ($15.70/DALYa), plastic surgery ($19.77/DALYa), and orthopedic surgery 368 ($21.41/DALYa) the most cost-effective specialties.

This was a very interesting study, shedding light on the provision of surgical care in a conflict area. Their conclusion that surgical care in conflict settings is not only morally just, but is also cost-effective and offer substantial economic benefits to help rebuild fragile economies is reasonable. The study design was robust, but inputs were inherently weaken without clinical outcomes data and relied heavily on informed estimates. However, sensitivity analyses were reassuring, and the authors correctly acknowledge prospectively assigning cost on a per patient basis would’ve made this a stronger study, using a micro-costing approach.

I would like the authors to further address:

what percentage of patients participate in the program in which patients are requested to offset costs with out-of-pocket expenditures from $1-$50 depending on type of care, although no patient is refused care for inability to pay?

**We appreciate this question, and have added this information in the text. Patient payments for care represented 1.4% of hospital revenues, with approx 50-60% of patients paying no out-of-pocket expenditures. All patient expenditures were accounted for in the hospital budget used for our calculations of the costs of care. In essence, patient expenditures offset a small amount of external financial resources, but did not affect the overall hospital budget and did not impact cost or cost-effectiveness calculations. All details of patient expenditures including rates for consultations and hospitalizations were added to the text.**

Additionally, the authors mention flat rates. What are these rates specifically? (“a flat rate for all consultations, a flat weekly rate for hospitalizations, and three different levels of payment for surgical procedures (minor, major, or complex)”).

**See above.**

Page 7 line 202 - missing verb “use/utilize” between “chose to” and “this extrapolation”

**We appreciate this catch of our grammatical error and have made this correction.**

6. PLOS authors have the option to publish the peer review history of their article (what does this mean?). If published, this will include your full peer review and any attached files.

Do you want your identity to be public for this peer review? If you choose “no”, your identity will remain anonymous but your review may still be made public.

For information about this choice, including consent withdrawal, please see our Privacy Policy.

Reviewer #1: No

Reviewer #2: No

[NOTE: If reviewer comments were submitted as an attachment file, they will be attached to this email and accessible via the submission site. Please log into your account, locate the manuscript record, and check for the action link "View Attachments". If this link does not appear, there are no attachment files.]

While revising your submission, please upload your figure files to the Preflight Analysis and Conversion Engine (PACE) digital diagnostic tool, https://pacev2.apexcovantage.com/. PACE helps ensure that figures meet PLOS requirements. To use PACE, you must first register as a user. Registration is free. Then, login and navigate to the UPLOAD tab, where you will find detailed instructions on how to use the tool. If you encounter any issues or have any questions when using PACE, please email PLOS at figures@plos.org. Please note that Supporting Information files do not need this step.
